# Advancements in laser technologies for skin rejuvenation: A comprehensive review of efficacy and safety

**DOI:** 10.1111/jocd.16514

**Published:** 2024-08-19

**Authors:** Diala Haykal, Hugues Cartier, David Goldberg, Michael Gold

**Affiliations:** ^1^ Centre Laser Palaiseau Palaiseau France; ^2^ Centre Médical Saint Jean Arras France; ^3^ Director of Cosmetic Dermatology and Clinical Research, Schweiger Dermatology Group Clinical Professor of Dermatology, Icahn School of Medicine at Mt. Sinai New York New York USA; ^4^ Gold Skin Care Center Tennessee Clinical Research Center Nashville Tennessee USA

**Keywords:** dermatology, efficacy, laser technology, safety, skin rejuvenation

## Abstract

**Introduction:**

Laser technology has fundamentally transformed the landscape of dermatology, offering nuanced solutions for skin rejuvenation and resurfacing. This paper aims to explore the spectrum of laser technologies, from ablative to non‐ablative and fractional lasers, their mechanisms, benefits, and tailored applications for diverse skin conditions. As we delve into the intricacies of each technology, we also consider the scientific advancements that have made these treatments safer and more effective, promising a new horizon in skin rejuvenation.

**Objective:**

This comprehensive analysis seeks to evaluate recent advancements in laser technology for skin rejuvenation, focusing on efficacy, safety, and patient satisfaction.

**Methods:**

The selection criteria for studies in this publication focused on recent, peer‐reviewed articles from the last 20 years, emphasizing advancements in laser technologies for skin rejuvenation. Our comprehensive review involved searches in PubMed, Cochrane, Scopus and Google Scholar using keywords like “skin rejuvenation,” “laser technology,” “efficacy,” “safety,” and “dermatology.” This approach focused on inclusion of recent research and perspectives on the efficacy and safety of laser treatments in the field of dermatology.

**Results:**

Our literature review reveals advancements in laser skin resurfacing technologies, notably fractional lasers for minimal downtime rejuvenation, ablative lasers for precise tissue vaporization, and non‐ablative lasers for coagulation effect promoting collagen with reduced recovery. Hybrid and picosecond lasers are highlighted for their versatility and effectiveness in addressing a wide array of skin concerns. The findings also emphasize the development of safer treatment protocols for ethnic skin, significantly reducing risks like hyperpigmentation and scarring, thus broadening the scope of effective dermatological solutions.

**Conclusion:**

This extensive review of advancements in laser technologies for skin rejuvenation underscores a remarkable evolution in dermatological treatments, offering an expansive overview of the efficacy, safety, and patient satisfaction associated with these interventions. Furthermore, the exploration of combination treatments and laser‐assisted drug delivery represents a frontier in dermatological practice, offering synergistic effects that could amplify the therapeutic benefits of laser treatments.

## 1 INTRODUCTION

In the dynamic field of dermatology, the introduction of laser technologies has marked a seminal shift, fundamentally transforming the approach to skin rejuvenation.^1^ Over the past few decades, groundbreaking advancements in laser technologies have not only broadened the therapeutic arsenal for skin rejuvenation but also established a new paradigm in treating various skin conditions.^2^ Laser treatments have significantly evolved from basic tools with limited uses to advanced devices capable of precise, targeted interventions. This evolution, driven by technological advancements and a deeper understanding of skin physiology, has allowed for an unprecedented level of treatment customization and safety.^3^ Lasers offer minimally invasive solutions that reduce recovery times and achieve outcomes previously thought unattainable, thereby revolutionizing cosmetic procedures and making skin rejuvenation more accessible to a wider patient population. The spectrum of laser technologies, ranging from ablative lasers that remove the outer layers of skin to reveal fresh, rejuvenated skin beneath, to non‐ablative fractional lasers (NAFL) that induce coagulation within the skin to stimulate collagen production and skin tightening without harming the skin's surface, addresses a diverse array of patient requirements.^4^ Furthermore, fractional lasers target microscopic zones of the skin to optimize results with minimal recovery time^5^ (Figure 1). A thorough understanding of the mechanisms behind laser skin resurfacing is essential for optimizing outcomes and ensuring safety. This includes analyzing how different wavelengths interact with skin tissues, the process of selective photothermolysis, and the body's healing response to thermal damage.^4^

**FIGURE 1:**
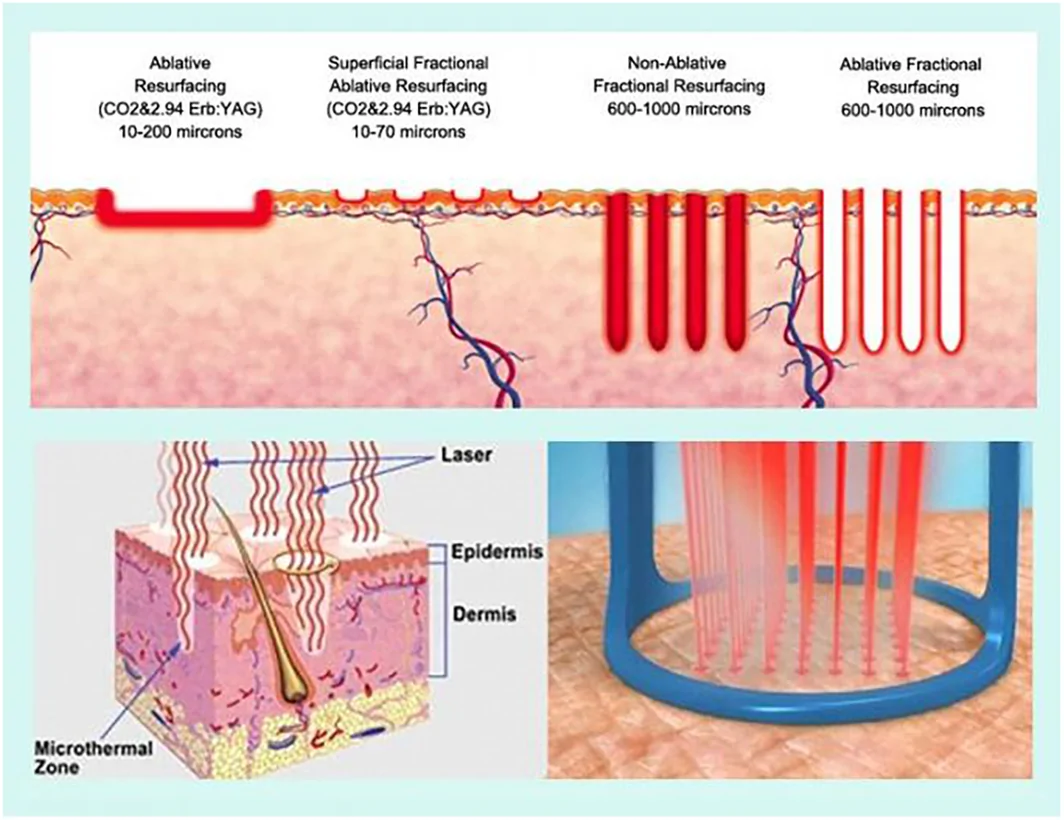
Ablative, non‐ablative, fractional resurfacing laser technologies.

The discussion covers the mechanisms, advantages, and personalized applications of laser technologies in skin resurfacing for diverse skin conditions, providing insights into the scientific advancements that have improved the safety and efficacy of these treatments.

## 2 METHODS

The present review contains materiel derived from articles published within the last 20 years, found using keywords like “skin rejuvenation,” “laser technology,” “efficacy,” “safety,” and “dermatology” on PubMed, Cochrane, Scopus, and Google Scholar. This meticulous approach ensured the inclusion of a broad spectrum of research and perspectives on the efficacy and safety of various laser skin resurfacing technologies. We systematically analyzed peer‐reviewed articles, clinical trial reports, and meta‐analyses, gathering extensive information that covers the evolution, mechanisms, benefits, and potential adverse effects associated with different laser technologies, including ablative, non‐ablative, fractional, hybrid, and picosecond lasers. Through this systematic methodology, we aim to provide an in‐depth examination of the laser technologies used in skin resurfacing, offering crucial insights into their efficacy and safety. Our goal is to observe the evolutionary process of laser skin resurfacing, pinpointing research gaps in order to propose future research.

## 3 RESULTS

Out of the initial 329 articles screened, 92 met our inclusion criteria based on relevance, study design, and recent advancements in laser technology for skin rejuvenation. The data gathered was systematically categorized into distinct sections. The categorization, listed as follows, was intended to compress the broad spectrum of lasers skin resurfacing technologies based on efficacy and safety:

### 3.1 Fractional lasers

Advancements in laser technologies for skin rejuvenation, particularly with fractional lasers, represent a significant leap forward in dermatological treatments. Fractional laser technology has revolutionized the way dermatologists address various skin concerns, offering patients remarkable results with minimal downtime and discomfort.^6^, ^7^, ^8^, ^9^

One of the most notable advancements is the ability of fractional lasers to deliver targeted energy to the skin in a fractional pixeled pattern, leaving surrounding tissue intact.^10^ This fractional approach promotes faster healing and reduces the risk of complications compared to traditional ablative lasers.^11^, ^12^ These lasers work by creating the microscopic columns of thermal damage in the skin, stimulating the body's natural healing response and triggering collagen production.^13^ As a result, fractional lasers can effectively treat a wide range of skin issues, including wrinkles, fine lines, acne scars, sun damage, and uneven pigmentation. Recent innovations in fractional laser technology have further improved treatment outcomes and patient experience. For instance, advancements in laser design have led to devices with customizable settings, allowing dermatologists to tailor treatments to each patient's specific needs and skin type.^7^, ^14^, ^15^ Furthermore, compared to the continuous fully systems, fractional lasers have become safer and more versatile. This versatility enables dermatologists to target different skin concerns and achieve optimal results with greater precision.^16^ Manstein and Laubach et al. confirm the significant impact of fractional photothermolysis on skin health, highlighting its role in enhancing dermal remodeling and reducing recovery times, thereby underscoring the transformative effects of these technologies on dermatological procedures.^4^, ^12^ This effect in turn, offers patients safe, effective, and customizable treatments for achieving smoother, younger‐looking skin. In not‐so‐far future, we can expect further refinements in fractional laser systems, leading to even better outcomes and expanding the possibilities for less and less invasive skin rejuvenation.

### 3.2 Ablative lasers

Research on CO_2_ laser mechanisms has clarified the process of selective photothermolysis, where the CO_2_ laser's intense light selectively targets water molecules in the skin.^17^ This action leads to the controlled vaporization and ablation of damaged tissue, causing a precise thermal injury that initiates a cascade of biological responses, including the generation of new collagen and the remodeling of the extracellular matrix.^18^ These changes result in enhanced skin texture, tone, and elasticity.^10^ Numerous clinical trials and observational studies have consistently shown the effectiveness of CO_2_ laser resurfacing for a variety of dermatological issues, such as photoaging, acne scars, dyschromias, and wrinkles.^19^, ^20^ Systematic reviews and meta‐analyses, synthesizing data from multiple studies, conducted by Miller, Verma and Clementoni et al. have confirmed significant improvements in both subjective and objective measures of skin quality, accompanied by high levels of patient satisfaction.^17^, ^18^, ^21^ Longitudinal studies have offered insights into the long‐term efficacy of CO_2_ laser resurfacing, indicating lasting improvements in skin texture and appearance.^20^, ^22^ Furthermore, when compared with other treatments like Er:YAG lasers, and chemical peels, CO_2_ laser resurfacing often shows superior efficacy, underscoring the need for personalized treatment plans based on individual patient needs and skin types.^23^, ^24^, ^25^ CO_2_ laser resurfacing has also become an integral part of combination treatment strategies, working in synergy with modalities like radiofrequency, micro‐needling, and topical agents to achieve better outcomes and comprehensively address complex skin concerns.^22^

Similarly, Er:YAG skin resurfacing treatments offer a distinct approach to improving skin texture and appearance, differing from CO_2_ laser treatments in several key aspects. Unlike CO_2_ lasers, which deliver deeper penetration and more extensive thermal effects conducive to pronounced collagen remodeling, Er:YAG lasers provide a more superficial action. This characteristic reduces the risk of post‐treatment complications such as hyperpigmentation, prolonged redness, or scarring, thereby shortening the recovery time.^26^, ^27^, ^28^ Alongside this, three studies conducted by Murray et al. showcase the 2910 nm laser's effectiveness in skin resurfacing for advanced photoaging, including full‐face and neck treatments. The first publication emphasizes its utility in single‐session treatments due to significant aesthetic improvements with minimal sessions. The subsequent studies widened the scope of these treatments via laser precision in both ablative fractional resurfacing and detailed histological analysis.^29^, ^30^, ^31^

### 3.3 Non‐ablative lasers

Non‐ablative lasers, including Nd:YAG, pulsed‐dye lasers (PDL), diode, thulium, and fractional erbium lasers, present alternative strategies for skin rejuvenation, characterized by reduced downtime and a lower risk of side effects.^32^, ^33^, ^34^ Specifically, NAFL adopt a focused approach, employing thermal energy to establish microthermal treatment zones (MTZs) within the skin. Although this precise heat application selectively damages the dermis, it preserves the epidermis, thus triggers the body healing processes and stimulates new collagen production.^35^ Consequently, this renewal of collagen enhances the skin's texture and firmness, significantly diminishing wrinkles, scars, and other imperfections.^15^, ^36^, ^37^ The minimal downtime associated with these technologies makes them a preferred choice for individuals in seek of improved skin appearance without invasive procedures.^38^ The use of NAFL has become a valued alternative to ablative lasers for skin resurfacing owing to benefits of enhanced skin texture and tone with minimal downtime and reduced risk of negative effects.^39^ These methods target the deeper layers of skin tissue avoiding noticeable damage to the surface, ideally suited for those desiring subtle enhancements. The efficacy of NAFL in promoting collagen production and skin remodeling leads to visibly smoother and tighter skin with minimal recovery time. Research supporting the effectiveness of these lasers indicates significant enhancements in skin texture, fine lines, and overall appearance, with a low likelihood of adverse reactions.^15^, ^37^, ^38^, ^40^, ^41^, ^42^ Patients generally report only mild discomfort during treatments and appreciate the short recovery period. Furthermore, the adaptability of NAFL allows for personalized treatment plans, with adjustable parameters to meet the unique needs and goals of each patient.

Beyond NAFL, other alternative non‐ablative lasers are Nd:YAG, diode, thulium, and PDL. Nd:YAG lasers penetrate deeper dermal layers to boost collagen production and improve skin elasticity. Whereas Diode and PDL lasers provide targeted solutions, particularly effective in treating vascular lesions and rosacea by focusing on blood vessels.^43^ The application of PDL in skin resurfacing, especially for treating various types of acne scars is primarily known for its success in managing vascular skin conditions. PDL also shows promise in addressing atrophic, hypertrophic, and keloidal scars by targeting oxyhemoglobin and minimizing erythema.^32^, ^44^, ^45^, ^46^ Specifically, Tao et al. highlight the efficacy of combining PDL with NAFL resurfacing for treating burn scars in Fitzpatrick phototype III patients, showcasing the advantages of multi‐modality treatments. Additionally, their research into the 1927 nm thulium laser demonstrates its effectiveness in epidermal burn scars hyperpigmentation targeting.^32^ Kono et al.'s comparison between intense pulsed light and long‐pulse pulsed dye laser reveals distinct advantages and suitability of each approach depending on specific skin rejuvenation needs.^44^ Tanghetti et al. highlight the versatility and efficacy of short‐pulsed lasers in treating a variety of skin concerns including tattoos, pigmented lesions, and scars.^45^ Lastly, Cohen and Geronemus underscore the enhanced efficacy of combined PDL and CO_2_ in the treatment of surgical scars.^46^ Adding to this, Khetarpal et al. present findings that emphasize the effectiveness of various advanced laser technologies, including ablative and NAFL, PDL, and CO_2_ lasers, in improving the texture and appearance of burn and traumatic scars.^47^ Ha et al. reveal that the thulium fiber, demonstrates potential for precise, controlled depth treatments, with its superficial tissue ablation capabilities compared to the CO and CO_2_ lasers.^48^ Vingan et al. focused on the specific benefits of the 1927 nm laser for diffuse dyspigmentation and actinic changes. Their findings support the laser's ability to even out skin tone and diminish signs of sun damage, contributing to a more uniform skin appearance.^49^ NAFL offers gentle skin resurfacing, leading to a smoother, firmer complexion.^50^, ^51^ However, it is essential to tailor the selection of laser and treatment protocol to an individual's skin type, scar characteristics, and therapeutic goals to ensure optimal outcomes with minimal side effects.^27^ To this end, NAFL supports versatile thus tailored treatments, enabling customization to align with individual patient preferences and objectives. (Table 1).

**TABLE 1:** Summary of several NAFL & AFL.

Nm	NAFL	Vascu	Pig	Rejuv	Fixed scar	Phototype >4
532 ms	No	Yes	Yes	Yes	No	No
532 ns	No	No	Yes	Yes	No	No
532 ps	Option	No	Yes	Yes	Yes frax	No
577 ms	Option	Yes	Yes	Yes	No	No
589 ms	Option	Yes	Yes	Yes	No	Yes
595 ms	No	Yes	Yes	Yes	No	No
694 ns	Option	No	Yes	Yes	No	No
755 ms	No	No	Yes	Yes	No	No
755 ps	Option	No	Yes	Yes	Yes	Yes
1064 sp		Yes	Yes	Yes	Yes	Yes
1064 ns	Option	No	Yes	Yes	Yes	Yes
1064 ps	Option	No	Yes	Yes	Yes	Yes
1064 lp	No	Yes	No	Yes	No	No
1319 nm	Option	No	Yes	Yes	Yes	Yes
1340 nm	Yes	No	No	Yes	Yes	Yes
1470 nm	Yes	No	Yes	Yes	Yes	Yes
IPL	No	Yes	Yes	Yes	Y/n	No
1540 nm	Yes	No	Yes	Yes	Yes	Yes
1550 nm	Yes	No	Yes	Yes	Yes	Yes
1565 nm	Yes	No	Yes	Yes	Yes	Yes
1570 nm	Yes	No	Yes	Yes	Yes	Yes
1927 nm	Yes	No	Yes	Yes	Yes	Yes

	AFL	Vascu	Pig	Rejuv	Fixed scar	Phototype >4
2910 nm	Option	No	Yes	Yes	Yes	Yes/no
2940 nm	Option	No	Yes	Yes	Yes	Yes/no
10 600 nm	Option	No	Yes	Yes	Yes	Yes/no

Abbreviations: AFL, ablative fractional laser; lp, long pulse (>5 ms); ms, millisecond; NAFL, non‐ablative fractional laser; nm, nanometer; ns, nanosecond (4 to 50); ps, picosecond (190–750); sp, short pulse (>0, 1 ms).

### 3.4 Hybrid fractional lasers

Skin resurfacing using hybrid lasers represents a significant advancement in cosmetic dermatology and aesthetic medicine, offering a versatile and effective approach to improving skin texture, tone, and overall appearance.^52^ This innovative treatment combines the benefits of different laser wavelengths in a single session, allowing for customized treatments that can address a variety of skin concerns with reduced downtime and enhanced outcomes.^53^

Hybrid laser systems typically integrate ablative and NAFL technologies. Ablative lasers work by removing the outer layers of damaged or aged skin, stimulating collagen production and making way for new, healthy skin to emerge. NAFL, on the other hand, penetrates deeper without removing the top layer of skin, heating up the underlying skin tissues to encourage collagen growth and skin tightening.^54^ By combining these technologies, hybrid lasers provide comprehensive skin rejuvenation, targeting everything from fine lines and wrinkles to acne scars and sun damage.^55^ One of the most lauded benefits of hybrid laser skin resurfacing is its ability to deliver noticeable improvements with less risk and downtime compared to traditional resurfacing methods. Patients often report less discomfort during the procedure and a quicker return to normal activities, thanks to the dual‐action approach that minimizes surface damage while maximizing the regenerative processes in the deeper layers of the skin.^5^ As demonstrated by Fusano et al., their study on hybrid fractional laser treatment for photodamaged facial skin rejuvenation, conducted 6 years after fractional CO_2_ treatments, highlights the sustained clinical outcomes and high patient satisfaction associated with these hybrid systems, confirming their efficacy in long‐term skin rejuvenation.^52^ As mentioned before, the versatility of hybrid lasers facilitates treatments tailored to specific needs and skin types of individuals. Waibel et al. indicates significant patient satisfaction and reduced recovery times to support photorejuvenation procedures.^54^ By leveraging the combined strengths of ablative and NAFL, hybrid lasers offer a compelling solution for individuals seeking to rejuvenate their skin with minimal downtime.

### 3.5 Picosecond lasers

The review by Haykal et al. provides an insightful overview of the current state of picosecond laser technology in cosmetic dermatology. It indicates that picosecond lasers are effective, not only for tattoo removal but also benign pigmented lesions, photodamage, melasma, and dermal remodeling.^56^ This expansion of applications highlights the versatility and advancement of picosecond laser technology in aesthetic dermatology. Bernstein et al. further demonstrate the efficacy of picosecond lasers on pigmented lesions and photodamage treatments.^57^ Furthermore, Punyaphat et al. show that fractional picosecond lasers equal effectiveness of fractional CO_2_ in addressing atrophic acne scars, supported by indications of tissue remodeling, while offering a safer profile.^58^

Moreover, the ultra‐short pulse duration of picosecond lasers provides treatment possibilities for various skin conditions effectively by substantial skin resurfacing; while minimizing thermal damage to adjacent tissues. The operational principle of these lasers is based on Laser‐Induced Optical Breakdown (LIOB), where focused laser energy generates a localized plasma field, leading to the formation of microcavitations; tiny gas bubbles that disrupt target cells with minimal heat production (Figures 2 and 3). This mechanism promotes significant therapeutic benefits without extensive thermal damage. Zhou et al. confirm that these updates on fractional picosecond lasers enhance skin texture and stimulate collagen production with recovery periods.^59^, ^60^ All in all, the versatility of picosecond lasers allows for their use in a variety of skin types and conditions, making them a valuable tool in aesthetic dermatology.^61^, ^62^

**FIGURE 2:**
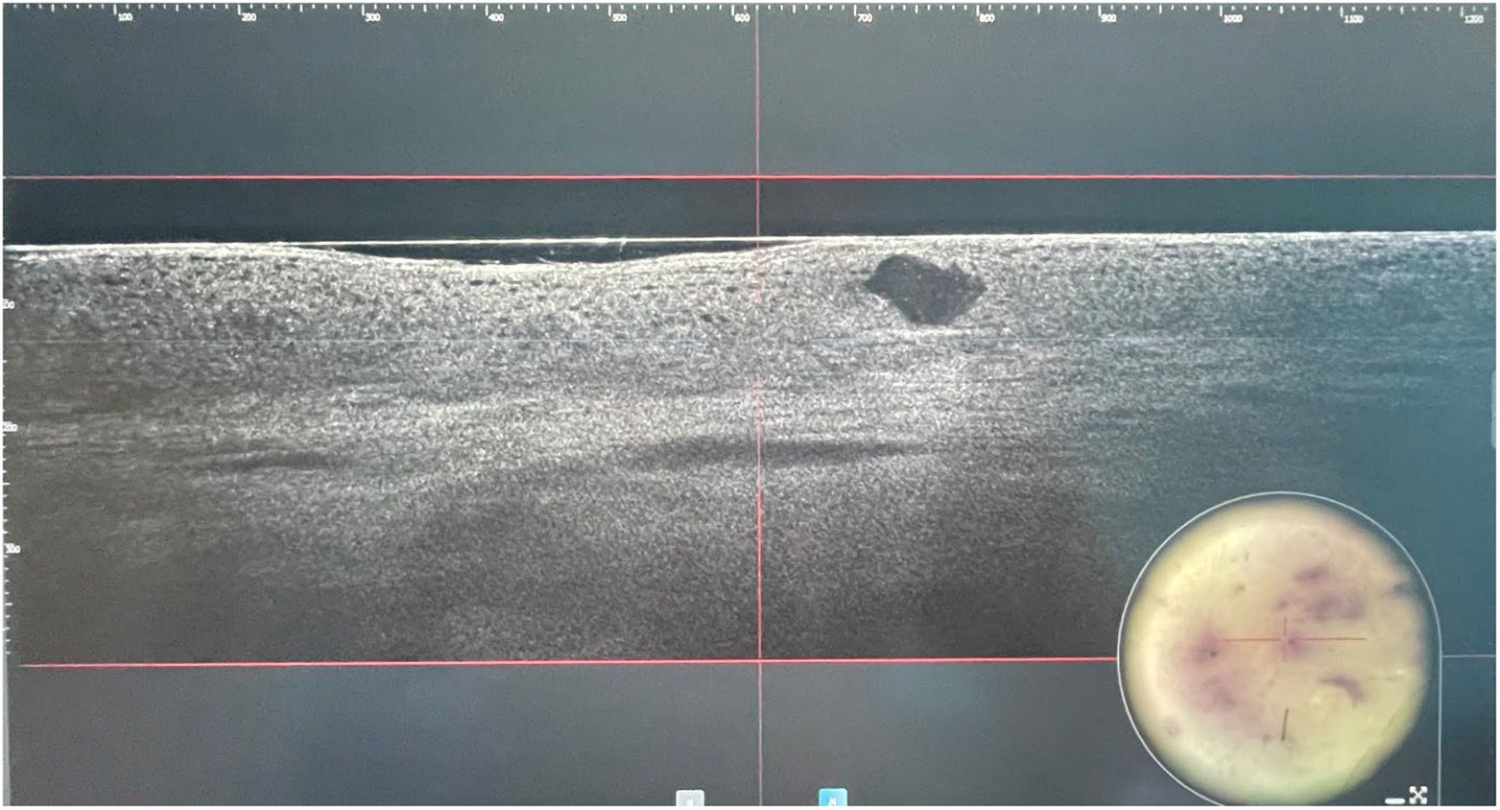
Laser‐induced optical breakdown in LC OCT.

**FIGURE 3:**
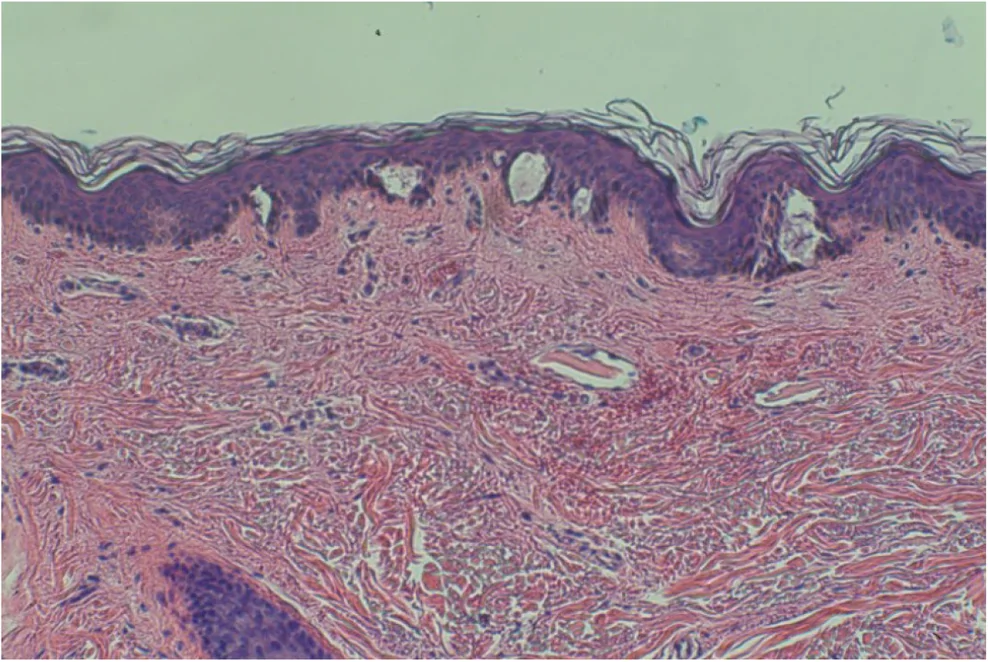
Laser‐induced optical breakdown in histology.

### 3.6 Laser technologies in ethnic skin rejuvenation

Laser technologies for ethnic skin rejuvenation have advanced, offering safer and more effective treatments for darker skin tones. These advancements minimize risks, effectively addressing the unique challenges of higher melanin content.^42^ Contemporary laser systems, including ND:YAG, picosecond, and fractionated lasers, have been specifically designed to minimize these risks.^41^, ^63^ They work by targeting the skin's deeper layers without damaging the surface, promoting collagen production, and treating various skin issues with minimal side effects.^42^, ^64^ This section underscores the importance of selecting the right laser technology and treatment parameters for optimal results, especially in treating acne scars, fine lines, wrinkles, textural irregularities and melasma in ethnic skin. Kaushik et al. have reviewed 48 articles which highlight the advancements and nuanced approach required for darker skin tones. The key factors for ensuring favorable outcomes include careful patient selection, setting realistic expectations, choosing the appropriate device, using conservative settings, and applying broad‐spectrum sunscreen to enhance safety and effectiveness.^42^ Fractionated laser technology, which creates microthermal treatment zones within the skin, allows for aggressive treatment of skin issues while leaving surrounding tissues intact. This, in turn, leads to quicker healing.^64^ Moreover, lasers such as the Nd:YAG, which operates at a wavelength that is less absorbed by melanin, and picosecond lasers, known for their short pulse duration minimizing thermal damage, have become popular choices for practitioners treating ethnic skin.^65^, ^66^, ^67^ Another aspect of laser treatment in ethnic skin rejuvenation is the pre‐treatment and post‐treatment care. It involves preparing the skin with melanin‐inhibitors to minimize the risk of hyperpigmentation and a thorough post‐treatment regimen that supports skin healing and maintenance of the treatment results.^68^

### 3.7 Safety and side effects

Ablative lasers, particularly with the advancement of fractional technology, have firmly established themselves as the gold standard for skin resurfacing procedures. This technology not only delivers significant clinical improvements but also minimizes side effects and shortens recovery times, making it an increasingly accessible option for a broader range of patients. As the safety of ablative resurfacing has improved over time, the emphasis on reducing complications and achieving optimal results hinges on meticulous patient selection and precise calibration of laser settings.^69^

Ensuring the safety of laser skin resurfacing requires understanding potential side effects and contraindications. Mild and transient side effects like redness, swelling, and pigmentary changes are common with both fractional ablative and NAFL. A comprehensive approach, including careful patient evaluation, selection, and post‐treatment care, is crucial for managing these effects. Practitioner expertise is vital for precise treatment. Initial evaluations and follow‐up care are essential to minimize complications, with stringent sun avoidance and recommended skincare products being key components. For patients with darker skin tones, selecting appropriate laser parameters is critical to prevent pigmentary changes. Advances in fractional and hybrid lasers have led to more precise energy delivery, enhancing safety, reducing recovery times, and decreasing the risk of adverse outcomes. As detailed by Agrawal et al., their review underscores these points, detailing how ablative skin resurfacing has evolved to include these newer, safer methods, providing valuable insights into clinical practices and outcomes.^69^ However, the call for ongoing research remains, particularly for individuals with darker complexions (skin types V–VI), to refine laser application techniques and expand the supportive evidence for these procedures. By strategically leveraging fractional lasers and deepening our comprehension of skin color variations, the field of dermatology can continue to progress, ensuring effective, inclusive, and safe treatments for a diverse array of patients.

### 3.8 Histology

Lasers and energy‐based devices (EBDs) serve as potent tools for skin resurfacing and rejuvenation, each employing unique mechanisms that target different skin structures. To use these devices safely and effectively, it is essential to understand the specific histological interactions between the laser and skin tissue.

Fractional picosecond lasers create intraepidermal and/or dermal vacuoles through LIOB, while NAFL create conical zones of coagulation within the epidermis and the upper dermis. Ablative lasers, in contrast, remove the stratum corneum extending down to the dermis. The histological changes induced by laser skin resurfacing treatments are profound and varied, depending on the type of laser employed. Ablative lasers offer immediate and noticeable results through significant epidermal ablation and dermal remodeling. In contrast, NAFL provide a gentler option, targeting the dermis to stimulate collagen production with minimal downtime. Fractional lasers merge these approaches, offering a compromise between efficacy and recovery time, while picosecond lasers present a novel method for skin rejuvenation, focusing on cellular disruption with minimal thermal damage. Each laser type possesses unique characteristics and benefits, tailored to meet diverse patient needs and treatment goals. Their targeted disruption promotes rapid healing and collagen production, facilitating effective skin remodeling with reduced recovery time and side effects as provided by Zhou, Kist and Taudorf et al.^59^, ^70^, ^71^ Understanding these histological changes is paramount for optimizing treatment outcomes and advancing the field of cosmetic dermatology.

#### 3.8.1 Epidermal and dermal responses

Traditional ablative lasers vaporize tissue uniformly, enhancing collagen density and structure, while fractional ablative lasers focus on ablating tissue in specific, targeted columns.^4^, ^28^ The micro‐fractional ablative skin resurfacing conducted by Dierickx et al. using novel erbium laser systems illustrates how these targeted approaches can significantly improve skin texture and healing time compared to more uniform ablation techniques.^28^ NAFL, though less invasive, still boost the turnover and thickness of the epidermis by creating microthermal zones, conical areas of coagulation that affect both the epidermis and the upper dermis gently and promote neocollagenesis without morphologically modifying the tissue, thus completely sparing the epidermis from damage^15^, ^72^ (Figure 1). Furthermore, the comparison of erbium:YAG and CO_2_ lasers in the resurfacing of facial rhytides by Khatri et al. reinforces the versatility and efficacy of these ablative technologies in targeting and restructuring the skin's deeper layers, leading to substantial improvements in the appearance of rhytides.^26^ Fractional picosecond lasers further advance these techniques by inducing the formation of intraepidermal and/or dermal vacuoles via LIOBs, leveraging photomechanical effects to renew the epidermis with minimal damage and ensure a rapid recovery^59^, ^73^ (Figures 2 and 3).

#### 3.8.2 Microvascular responses

Skin resurfacing laser treatments can have profound effects on the skin's vascular architecture. The thermal energy from lasers can coagulate blood vessels, reducing redness and targeting vascular lesions, thereby improving overall skin tone. Additionally, the heat can seal blood vessel segments within the MTZs, disrupting blood flow and reducing telangiectasias. This effect suggests fractional photothermolysis could be a new method for targeting blood vessels when their distribution matches the thermal damage pattern. The microvascular responses vary with the laser type and settings, with certain wavelengths being more effective for vascular targeting. According to Tull and Raza, the subsequent vascular remodeling supports skin rejuvenation, contributing to a more uniform complexion, as laser treatments can lead to increased uniformity and clarity in skin appearance by effectively reducing vascular irregularities.^74^ Azzam et al. further elaborated on this by showing that fractional carbon dioxide lasers not only improve the appearance of hypertrophic scars and keloids but also positively impact the vascular components within these scar tissues, leading to reduced scar vascularity and an improved aesthetic outcome.^75^

#### 3.8.3 Immune responses

Laser resurfacing can also modulate the skin's immune response. The controlled injury inflicted by lasers, particularly ablative and fractional types, activates the immune system's wound healing cascade. This includes the recruitment of immune cells to the site of injury, promoting the removal of damaged tissue and facilitating the repair process. The thermal stress from the laser treatment can enhance the skin's resilience by stimulating an immune‐mediated repair mechanism, which plays a crucial role in the rejuvenation process.^76^ Guo et al. reported that fractional CO_2_ laser treatment plays a crucial role in skin rejuvenation by activating the immune system. This activation results in the secretion of numerous cytokines, aiding in the transformation of treated areas towards normal skin conditions. Additionally, the treatment facilitates skin barrier regulation, collagen rearrangement, and enhancement of local microvascular circulation, thus acting as a balancer in skin reconstruction.^77^ This immune activation is integral to the remodeling of both the epidermal and dermal layers, ultimately contributing to the skin's improved appearance and health.

### 3.9 Long‐term effects

Considering long‐term histological effects, repeated laser sessions have been shown to further enhance skin rejuvenation outcomes. Alexiades, Tan, Hsiao et Majid et al. revealed that multiple treatments with both ablative and NAFL lead to cumulative improvements in collagen density and skin elasticity over time.^38^, ^78^, ^79^, ^80^ This suggests that a tailored, multi‐session laser treatment plan could be beneficial in achieving optimal long‐term aesthetic results. These histological changes underscore the efficacy of laser skin resurfacing in improving skin texture and appearance.^67^ By precisely targeting and manipulating the skin's structural components at the microscopic level, this procedure offers a powerful solution for a variety of skin concerns, from photoaging to scarring, with outcomes that are grounded in the intricate details of skin histology.

### 3.10 Patient satisfaction

This review consistently emphasizes high levels of patient satisfaction, highlighting the significant impact of these treatments on improving skin conditions and quality of life. Nearly all studies demonstrate that patients report substantial satisfaction due to visible improvements in skin texture, reduction in wrinkles, scars, and pigmentation, and overall rejuvenation effects.^29^, ^50^, ^66^, ^81^, ^82^ This satisfaction stems not only from the visible cosmetic improvements but also from the relatively short recovery periods, the minimally invasive nature of the procedures, and the few side effects experienced. For instance, Mani et al. detailed the successful use of a combination of fractional and full spot ablative 2940 nm erbium laser for full‐face skin resurfacing, noting significant patient satisfaction with their outcome.^81^ Similarly, Knight et al. reported positive results from a sequential approach using intense pulsed light and non‐ablative fractionated laser resurfacing, which was particularly effective in Fitzpatrick skin type II–IV patients.^82^ Furthermore, Murray et al. explored the efficacy of a novel ablative fractional 2910 nm laser for treating advanced photoaging on the full face and neck, emphasizing remarkable improvements in skin quality and high patient satisfaction rates.^29^

Despite the high satisfaction rates, the literature emphasizes the importance of comprehensive pre‐treatment consultations and setting realistic expectations to sustain high levels of patient satisfaction with laser resurfacing outcomes, ensuring that results align closely with patient expectations.^83^, ^84^, ^85^

## 4 DISCUSSION

The introduction of various laser technologies in skin resurfacing has significantly expanded treatment options for diverse skin disorders, offering exceptional accuracy and effectiveness. Various laser technologies, such as CO_2_ and Er:YAG, can induce surface‐level vaporization using ablative lasers. NAFL, on the other hand, focus on activating collagen beneath the skin. Fractional lasers employ a pixelated approach to minimize downtime and side effects. Furthermore, the emergence of hybrid laser systems that combine the advantages of both ablative and non‐ablative technology highlights the ever‐evolving character of this discipline, providing customized methods for complete skin rejuvenation. This selective approach allows for a faster recovery and reduces the side effects associated with treatment. The literature corroborates our findings, with numerous comparative studies, clinical case reports, and comprehensive reviews highlighting the distinct mechanisms and outcomes associated with ablative, and NAFL.

It is also noteworthy to highlight the synergistic combination of laser skin resurfacing technologies with pharmacological agents or cosmeceuticals. It represents a significant advancement, enhancing therapeutic outcomes beyond what could be achieved by either modality alone.^86^ Lasers, by creating micro‐channels in the skin, serve as a conduit for increased penetration of topical drugs or cosmeceuticals, thereby amplifying their efficacy.^40^, ^71^, ^87^, ^88^, ^89^ This method, often referred to as laser‐assisted drug delivery, leverages the precision and controlled damage of laser treatments to facilitate deeper absorption of therapeutic agents, effectively targeting underlying skin issues at a cellular level. Several research support the efficacy of this approach, such as those conducted by Zamanian et al. which have demonstrated enhanced outcomes in treating conditions like infra‐orbital dark circles when fractional CO_2_ laser was in combination therapy with micro‐needling and topical TCA.^22^ Similarly, Waibel et al. have highlighted the potential of combining laser treatments with corticosteroids to manage keloids and hypertrophic scars, showing a reduction in scar thickness and improved aesthetic results.^90^ This integrated treatment strategy opens new avenues for treating a range of skin conditions more effectively. By leveraging the dual benefits of laser technology and the potent action of pharmaceuticals and cosmeceuticals, practitioners can offer tailored, more effective treatments, setting a new standard in cosmetic and therapeutic dermatology.

Another challenging concern in laser skin resurfacing is the effectiveness of cooling systems in mitigating pain and reducing side effects. Cooling systems are vital for reducing pain and side effects by managing the thermal impact of treatments. These technologies enhance patient safety, comfort, and satisfaction by minimizing discomfort and preventing burns or scarring. Despite significant advancements, ongoing research is needed to further optimize cooling systems, ensuring treatment efficacy and the highest standards of patient care.^91^, ^92^ The continuous advancement of laser technologies is expanding possibilities in skin resurfacing by exploring novel wavelengths, delivery systems, and treatment protocols. This progress aims to provide individualized and highly effective skin resurfacing solutions. The complexity of laser skin resurfacing involves clinical, technological, and ethical factors. Meticulous patient assessment is essential to determine the most appropriate laser method, ensuring safety and satisfaction. This holistic approach is critical for advancing therapeutic technology and improving patient care and outcomes.

### 4.1 Limitations

This review extensively explores the advancements in laser technologies for skin rejuvenation, however it has some limitations to be considered. We acknowledge that most research focuses on short‐term results, leaving a gap in understanding about the long‐term effects. The studies included exhibit considerable variability in design, methodologies, laser parameters, and outcome measures, which can make it difficult to synthesize data and draw comprehensive conclusions that are universally applicable. Additionally, there is a notable underrepresentation of individuals with darker skin types in the analyzed studies, which may limit the applicability of the findings to the global population. While this analysis encompasses the most common systems and EBDs, it is important to note that some wavelengths may not have been included; however, the most prevalent ones have been adequately addressed. Furthermore, with the rapid pace of technological advancements in the field, some findings may quickly become outdated, underscoring the need for ongoing research to keep pace with innovations and ensure the relevance and reliability of information regarding laser skin rejuvenation techniques.

## 5 CONCLUSION

In conclusion, laser skin resurfacing offers a comprehensive and advanced method for skin rejuvenation, addressing various conditions with tailored treatments. Ablative lasers like CO_2_ and Er:YAG are effective for deeper skin issues but have greater side effects and longer recovery times. Non‐ablative technologies promote collagen production and skin tightening with minimal surface damage, ideal for milder concerns and minimal downtime. Fractional lasers combine the benefits of both, balancing healing time and complication reduction. Careful patient selection based on skin type and goals ensures safe and successful outcomes. Future advancements in non‐ablative laser technology are expected to improve targeting precision and tissue penetration. Studies are exploring combinations with other treatments to enhance results and address more skin issues. Continued innovation and research will likely boost treatment effectiveness, expand options, and improve safety, particularly for individuals with darker skin tones, broadening the appeal of laser skin rejuvenation.

## AUTHOR CONTRIBUTIONS

D.H. had the concept for the survey and performed the writing with assistance from H.C., D.G., and M.G.. All authors read and approved the final manuscript.

## CONFLICT OF INTEREST STATEMENT

The author declares no conflicts of interest.

## ETHICS STATEMENT

This study did not involve any human participants, human data, or animals and therefore did not require ethical approval.

## Data Availability

The data that support the findings of this study are available from the corresponding author upon reasonable request.
